# Supplementary material for: Analysis of PPARγ Signaling Activity in Psoriasis
Source: Int J Mol Sci. 2021 Aug 10;22(16):8603. doi: 10.3390/ijms22168603 (PMC8395241; doi:10.3390/ijms22168603)
Supplement: Supplementary file 1 [file ijms-22-08603-s001.zip › Supplemental materials_Analysis of PPARg signaling activity in psoriasis/Pathway models/Models images and html files/Differentiation of psoriatic T cells/14219.html]

IL27RA


# Protein IL27RA

|  |  |
| --- | --- |
| URN | urn:agi-llid:9466 |
| Total Entities | 0 |
| Connectivity | 291 |
| Name | IL27RA |
| Description | interleukin 27 receptor, alpha |
| Notes | In mice, CD4+ helper T-cells differentiate into type 1 (Th1) cells, which are critical for cell-mediated immunity, predominantly under the influence of IL12. Also, IL4 influences their differentiation into type 2 (Th2) cells, which are critical for most antibody responses. Mice deficient in these cytokines, their receptors, or associated transcription factors have impaired, but are not absent of, Th1 or Th2 immune responses. This gene encodes a protein which is similar to the mouse T-cell cytokine receptor Tccr at the amino acid level, and is predicted to be a glycosylated transmembrane protein. [provided by RefSeq, Jul 2008] |
| Class | Receptor |

---

|  |  |
| --- | --- |
| Pathway | Th1-Cell Differentiation |
|  | AHR Signaling in Tr1 Cells Function |
|  | Eosinophil Survival by Cytokine Signaling |
|  | Th1-Cells Activation and Proliferation in Atherosclerosis |
|  | Lymphocyte Mediated Myocardial Injury in Myocarditis |
|  | Atopic Dermatitis |
|  | T-Cells Differentiation Block in Psoriasis |
|  | T-Cell Cytotoxic Response against Melanocytes in Vitiligo |
|  | CD4+T-Cell Response in Celiac Disease |
|  | Th1-Cell Activation in Crohn's Disease |
|  | IL27R -> STAT Signaling |
|  | PPAR Psoriasis |
|  | 1\_Differentiation of psoriatic T cells |
|  | Differentiation of psoriatic T cells |

---

|  |  |
| --- | --- |
| MedScan ID | 9466 |

---

|  |  |
| --- | --- |
| LocusLink ID | 9466 |
|  | 50931 |
|  | 288905 |

---

|  |  |
| --- | --- |
| Alias | T-cell cytokine receptor |
|  | type I T-cell cytokine receptor |
|  | IL27RA |
|  | WSX I |
|  | IL-27 receptor subunit alpha |
|  | cytokine receptor WSX-1 |
|  | interleukin IL27RA |
|  | TCCR |
|  | IL-27R subunit alpha |
|  | cytokine receptor-like 1 |
|  | WSX1 |
|  | UNQ296/PRO336 |
|  | LOC234540 |
|  | interleukin-27 receptor subunit alpha |
|  | WSX1/IL-27R |
|  | cytokine receptor family, class 1 (WSXWS), member 1 |
|  | wsxws |
|  | IL-27R-alpha |
|  | IL-27R/WSX1 |
|  | zcytor1 |
|  | T-cell cytokine receptor type 1 |
|  | interleukin 27 receptor, alpha |
|  | CRL1 |
|  | IL27R |
|  | IL-27RA |
|  | class I cytokine receptor |
|  | WSX-1 |
|  | IL-27R |
|  | T cell cytokine receptor |
|  | T cell cytokine receptor; cytokine receptor family, class 1 (WSXWS), member 1 |
|  | interleukin IL27R |
|  | CRL I |
|  | Il27ra\_predicted |

---

|  |  |
| --- | --- |
| GO ID | 0019955 |
|  | 0004896 |
|  | 0045509 |
|  | 0004888 |
|  | 0007166 |
|  | 0050830 |
|  | 0006955 |
|  | 0070106 |
|  | 0070757 |
|  | 2000408 |
|  | 2000317 |
|  | 1905077 |
|  | 1900165 |
|  | 1904468 |
|  | 0002829 |
|  | 0002827 |
|  | 0032729 |
|  | 0048302 |
|  | 0009897 |
|  | 0005887 |
|  | 0005886 |
|  | 0043235 |
|  | 0002692 |
|  | 0016021 |
|  | 0016020 |
|  | 0004872 |
|  | 0007165 |

---

|  |  |
| --- | --- |
| KEGG ID | hsa:9466 |
|  | mmu:50931 |
|  | rno:288905 |

---

|  |  |
| --- | --- |
| Organism | Homo sapiens {Organism urn:agi-taxid:9606} |
|  | Mus musculus {Organism urn:agi-taxid:10090} |
|  | Rattus norvegicus {Organism urn:agi-taxid:10116} |
|  | Mus musculus |
|  | Homo sapiens |
|  | Rattus norvegicus |

---

|  |  |
| --- | --- |
| Mouse chromosome position | 8 |

---

|  |  |
| --- | --- |
| OMIM ID | 605350 |

---

|  |  |
| --- | --- |
| Rat chromosome position | 19q11 |

---

|  |  |
| --- | --- |
| Hugo ID | 17290 |
|  | HGNC:17290 |

---

|  |  |
| --- | --- |
| Human chromosome position | 19p13.12 |
|  | 19p13.11 |

---

|  |  |
| --- | --- |
| Swiss-Prot Accession | Q6UWB1 |
|  | Q6UWB1.2 |
|  | O70394 |
|  | Q3UZN3 |
|  | O70394.2 |
|  | D4A6K5 |
|  | A0A0G2JW79 |
|  | A0N0L1 |
|  | O60624 |
|  | E9QNY4 |

---

|  |  |
| --- | --- |
| PIR ID | JW0047 |

---

|  |  |
| --- | --- |
| GenBank ID | NC\_000019 |
|  | NM\_004843 |
|  | NP\_004834 |
|  | NW\_021160022 |
|  | AC022098 |
|  | CH471106 |
|  | EAW84389 |
|  | EAW84390 |
|  | EF064719 |
|  | ABK41902 |
|  | FB507086 |
|  | CAV32862 |
|  | HH829741 |
|  | CBX32720 |
|  | AF053004 |
|  | AAC39755 |
|  | AF106912 |
|  | AAG26090 |
|  | AF265242 |
|  | AAG27297 |
|  | AI983115 |
|  | AW298502 |
|  | AY358876 |
|  | AAQ89235 |
|  | BC028003 |
|  | AAH28003 |
|  | Q6UWB1 |
|  | NC\_000074 |
|  | NM\_016671 |
|  | NP\_057880 |
|  | XM\_011248437 |
|  | XP\_011246739 |
|  | XR\_001778452 |
|  | XM\_011248438 |
|  | XP\_011246740 |
|  | AC156028 |
|  | CH466525 |
|  | EDL10927 |
|  | EDL10928 |
|  | AF053005 |
|  | AAC40121 |
|  | AF265243 |
|  | AAG27298 |
|  | AK040457 |
|  | AK133753 |
|  | BAE21823 |
|  | BC032878 |
|  | AAH32878 |
|  | BF019826 |
|  | O70394 |
|  | NC\_005118 |
|  | XM\_006255227 |
|  | XP\_006255289 |
|  | XM\_017601191 |
|  | XP\_017456680 |
|  | XM\_006255228 |
|  | XP\_006255290 |
|  | NM\_001105943 |
|  | NP\_001099413 |
|  | AC\_000087 |
|  | AABR07043168 |
|  | AABR07043169 |
|  | AAHX01097744 |
|  | CH473972 |
|  | EDL92242 |
|  | NC\_018930 |
|  | AMYH02035597 |
|  | AMYH02035598 |
|  | DQ891051 |
|  | ABM81977 |
|  | DQ894230 |
|  | ABM85156 |
|  | AC\_000030 |
|  | AAHY01072702 |
|  | AAHY01072703 |
|  | AC\_000151 |
|  | ABBA01029695 |
|  | ABBA01029696 |
|  | ABBA01029697 |
|  | XR\_387757 |
|  | XM\_006531181 |
|  | XP\_006531244 |
|  | XM\_006531180 |
|  | XP\_006531243 |
|  | NT\_011295 |
|  | NW\_001838483 |
|  | NW\_927195 |
|  | AC\_000062 |
|  | NT\_078575 |
|  | NW\_001030904 |
|  | Q3UZN3 |
|  | NW\_047534 |
|  | NW\_001084744 |

---

|  |  |
| --- | --- |
| Swiss-Prot ID | I27RA\_HUMAN |
|  | I27RA\_MOUSE |
|  | D4A6K5\_RAT |
|  | E9QNY4\_MOUSE |
|  | Q3UZN3\_MOUSE |
|  | A0N0L1\_HUMAN |

---

|  |  |
| --- | --- |
| Cell Localization | Membrane |

---

|  |  |
| --- | --- |
| Ensembl ID | ENSG00000104998 |
|  | ENSP00000263379.1 |
|  | ENST00000263379.4 |
|  | ENSMUSG00000005465 |
|  | ENSMUSP00000005601.7 |
|  | ENSMUST00000005601.8 |
|  | ENSRNOG00000005747 |
|  | ENSRNOP00000007726.5 |
|  | ENSRNOT00000007726.5 |
|  | ENSRNOP00000069777.1 |
|  | ENSRNOT00000085630.1 |
|  | ENST00000263379.3 |
|  | ENSP00000263379 |
|  | ENST00000263379 |
|  | ENSMUSP00000005601 |
|  | ENSMUST00000005601 |
|  | ENSRNOP00000007726 |
|  | ENSRNOT00000007726 |
|  | ENSRNOP00000069777 |
|  | ENSRNOT00000085630 |

---

|  |  |
| --- | --- |
| MGI ID | MGI:1355318 |
|  | 1355318 |

---

|  |  |
| --- | --- |
| RGD ID | 1305029 |

---

|  |  |
| --- | --- |
| Unigene ID | Mm.38386 |
|  | Hs.352155 |
|  | Rn.47680 |
|  | Hs.132781 |

---

|  |  |
| --- | --- |
| Homologene ID | 3562 |

---

|  |  |
| --- | --- |
| Shape | Stick-vertex |

---

|  |  |
| --- | --- |
| IPI ID | IPI00006774 |
|  | IPI00117451 |
|  | IPI00371645 |
|  | IPI00988016 |

---
